# Impact of an Educational Comic to Enhance Patient-Physician–Electronic Health Record Engagement: Prospective Observational Study

**DOI:** 10.2196/25054

**Published:** 2021-04-28

**Authors:** Maria A Alkureishi, Tyrone Johnson, Jacqueline Nichols, Meera Dhodapkar, M K Czerwiec, Kristen Wroblewski, Vineet M Arora, Wei Wei Lee

**Affiliations:** 1 Department of Academic Pediatrics University of Chicago Chicago, IL United States; 2 Department of Internal Medicine University of California, San Francisco San Francisco, CA United States; 3 Department of Obstetrics and Gynecology University of Washington Seattle, WA United States; 4 Yale University School of Medicine New Haven, CT United States; 5 Center for Medical Humanities & Bioethics Feinberg School of Medicine Northwestern University Chicago, IL United States; 6 Department of Public Health Sciences University of Chicago Chicago, IL United States; 7 Department of Medicine University of Chicago Chicago, IL United States

**Keywords:** electronic health records, patient, comic, education, engagement

## Abstract

**Background:**

Electronic health record (EHR) use can impede or augment patient-physician communication. However, little research explores the use of an educational comic to improve patient-physician-EHR interactions.

**Objective:**

To evaluate the impact of an educational comic on patient EHR self-advocacy behaviors to promote patient engagement with the EHR during clinic visits.

**Methods:**

We conducted a prospective observational study with adult patients and parents of pediatric patients at the University of Chicago General Internal Medicine (GIM) and Pediatric Primary Care (PPC) clinics. We developed an educational comic highlighting EHR self-advocacy behaviors and distributed it to study participants during check-in for their primary care visits between May 2017 and May 2018. Participants completed a survey immediately after their visit, which included a question on whether they would be interested in a follow-up telephone interview. Of those who expressed interest, 50 participants each from the adult and pediatric parent cohorts were selected at random for follow-up telephone interviews 8 months (range 3-12 months) post visit.

**Results:**

Overall, 71.0% (115/162) of adult patients and 71.6% (224/313) of pediatric parents agreed the comic encouraged EHR involvement. African American and Hispanic participants were more likely to ask to see the screen and become involved in EHR use due to the comic (adult *P*=.01, *P*=.01; parent *P*=.02, *P*=.006, respectively). Lower educational attainment was associated with an increase in parents asking to see the screen and to be involved (ρ=−0.18, *P*=.003; ρ=−0.19, *P*<.001, respectively) and in adults calling for physician attention (ρ=−0.17, *P*=.04), which was confirmed in multivariate analyses. Female GIM patients were more likely than males to ask to be involved (median 4 vs 3, *P*=.003). During follow-up phone interviews, 90% (45/50) of adult patients and all pediatric parents (50/50) remembered the comic. Almost half of all participants (GIM 23/50, 46%; PPC 21/50, 42%) recalled at least one best-practice behavior. At subsequent visits, adult patients reported increases in asking to see the screen (median 3 vs 4, *P*=.006), and pediatric parents reported increases in asking to see the screen and calling for physician attention (median 3 vs 4, *P*s<.001 for both). Pediatric parents also felt that the comic had encouraged them to speak up and get more involved with physician computer use since the index visit (median 4 vs 4, *P*=.02) and that it made them feel more empowered to get involved with computer use at future visits (median 3 vs 4, *P*<.001).

**Conclusions:**

Our study found that an educational comic may improve patient advocacy for enhanced patient-physician-EHR engagement, with higher impacts on African American and Hispanic patients and patients with low educational attainment.

## Introduction

Electronic health record (EHR) use in clinical care has become the norm in the United States [1-3]. Studies on the impact of EHR use have found that certain physician behaviors (eg, poor eye contact, long silences) may lead to decreased patient satisfaction with the patient-physician relationship and communication [4-11]. While studies show there are certain patient-centered care behaviors that can positively impact patient satisfaction and health outcomes, with Table 1 serving as a model for incorporating many evidence-based behaviors, physicians are faced with the challenge of staying focused on their patients while efficiently navigating the EHR during clinical encounters [4,6,12-20].

In a 2016 study on patient perceptions of physician EHR use in an academic primary care practice, patients were dissatisfied when physicians appeared more focused on the computer than on them and frustrated with lack of transparency and poor body positioning, which contributed to perceptions of decreased quality of care [7]. While best practices to promote patient-centered EHR use have been identified, most physicians and patients are unaware of these strategies to improve patient-physician-EHR communication [6,12,20-26].

Educational comics have emerged as an innovative way to promote patient education and engagement in a variety of clinical settings including pediatric, gynecology, radiation oncology, neurology, and endocrine practices [27-34]. Despite these findings, to our knowledge, no studies have looked at using educational comics to promote patient-centered EHR use in academic primary care practices. Furthermore, prior studies have found that Black and Hispanic patients and those with lower educational attainment level experience increased health care disparities, which in turn may result in poorer health outcomes [35-47]. As such, we aim to assess the impact of an educational comic on patient self-advocacy behaviors to enhance patient engagement with the EHR and to determine if there are variable impacts of the comic on different patient demographic variables such as ethnicity and education attainment level.

**Table 1:** HUMAN LEVEL—10 tips to enhance patient-centered electronic health record use [20].

Initial	Tip	Description
H	*H*onor the “Golden Minute”	Make the start of the visit completely *technology-free*. Greet the patient, start with *their* concerns, and establish an *agenda* for the visit *before* engaging technology.
U	*U*se the “Triangle of Trust”	Create a *triangle configuration* that puts you, the patient, and the computer screen at each of the three corners. This allows you to look at both the patient and screen without shifting your body position, and also enables *shared* screen viewing.
M	*M*aximize patient interaction	Encourage patient *interaction*. Pause for questions and clarification. Allow time for questions and to verify understanding.
A	*A*cquaint yourself with chart	Review the chart *before* you enter the room to prepare, inform, and *contextualize* your visit.
N	*N*ix the screen	When discussing *sensitive* information, *completely disengage* from the EHR^a^ (look at the patient, turn away from screen, take hands off keyboard, etc).
L	*L*et the patient look on	*Share* things on the screen with your patients.
E	*E*ye contact	Maintain *eye contact* with patients as much as possible. Treat patient encounters as you would a conversation with friends or family members.
V	*V*alue the computer	Praise the *benefits* of the EHR and take advantage of opportunities to use technology as a tool to *engage* patients (pull up lab result to review together, utilize graphics, etc).
E	*E*xplain what you’re doing	Be *transparent* about everything you do. Avoid long silences, aim for conversational EHR use by explaining what you are doing as you are doing it.
L	*L*og off	At the end of the visit, *log off* of the patient’s chart while they are still in the exam room. This reassures patients that their medical information is *secure*.

^a^EHR: electronic health record.

## Methods

### Setting and Participants

The study was conducted at the University of Chicago’s General Internal Medicine (GIM) and Pediatric Primary Care (PPC) clinics between May 2017 and May 2018. Adult GIM patients and pediatric parents who were scheduled to see faculty physicians were approached by trained research assistants in the waiting room and verbally consented to participate in the study. Inclusion criteria included ability to consent and English proficiency. GIM and PPC faculty physicians were given information about the study at their respective section meetings and via email communications, and all consented to having their patients participate. Of note, the ergonomic room layout in both clinics is such that the screen is usually not easily viewed by the patient unless it or the chairs in the room are moved to encourage shared viewing.

### Comic Development

**Figure 1:**
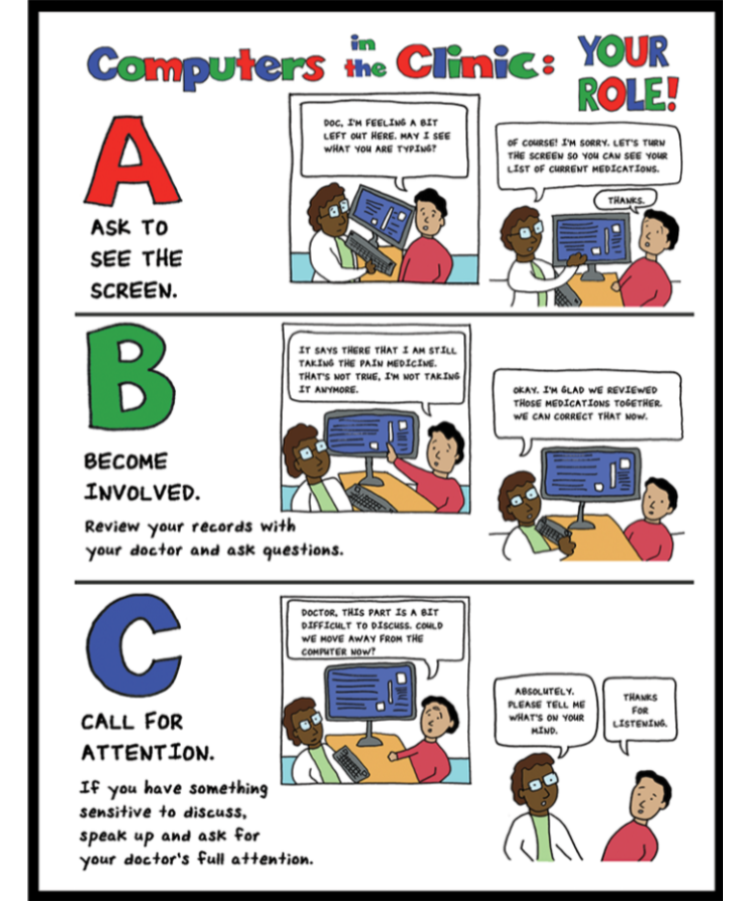
Patient EHR self-advocacy comic. The educational comic was given to adult patients and parents of pediatric patients when registering for their clinic visits to encourage EHR self-advocacy behaviors and engagement. EHR: electronic health record. © Alkureishi ML, Czerwiec MK, Arora V, Lee WW and the Arnold P. Gold Foundation.

The educational comic (Figure 1), “Computers in the Clinic: Your Role,” was developed by the authors (MAA, WWL, VMA, MKC) based on a literature review of the impact of EHR use on patient-physician communication [4-6,8,9]. The comic was drawn by author MKC, a practicing nurse with experience in designing educational comics for patient education interventions. It highlights three patient self-advocacy behaviors aimed at improving patient EHR engagement: (1) A for “Ask to see the screen” to promote screen sharing, (2) B for “Become involved with your doctor’s use of the computer” to encourage patient-physician-EHR interaction and patient education, and (3) C for “Call for attention” to encourage patients to speak up if they feel their physician is distracted by the EHR.

### Postvisit Survey and Telephone Interview Script Development

Using findings from a literature review, a 33-item postvisit survey was developed containing open-ended and Likert scale questions to assess the comic’s impact on patient (1) self-advocacy behaviors for more engaging and meaningful patient-physician-EHR interactions, (2) satisfaction with physician EHR use, and (3) perceptions of physician communication at the current visit compared to patient recollections of communication with the same provider at prior visits [4-6,8,9] (Multimedia Appendix 1). Studies have shown that patient self-report is a reasonable method of assessing whether educational interventions improve subsequent behaviors and self-advocacy [48-52]. Furthermore, we wanted to directly ask patients what they thought about the patient-physician-EHR interaction and impacts of the comic on their behavior and perceptions, rather than use an observer or their clinician’s perceptions as an indirect proxy.

A semistructured telephone interview script was developed to assess (1) patient recall of the comic and (2) impact of the comic on patient perceptions and self-advocacy behaviors and EHR engagement at subsequent physician visits (Multimedia Appendix 2). The interview script contained 6 5-point Likert-style questions to assess patient perceptions of the comic and impact on behaviors since the index visit (eg, “The comic encouraged me to speak up and get more involved with the computer at my subsequent visits with my doctor.”) as well as open-ended questions to prompt patient responses (eg, “Can you give me some examples of how you’ve asked to get more involved with your doctor’s use of the computer during clinic visits?”).

### Intervention

The hypothesis for our study was that more than 50% of respondents would agree that the comic made them get more involved with the computer (null hypothesis: ρ=50% vs alternative hypothesis: ρ>50%). Our calculations assumed 80% power and one-sided exact binomial test with α=.025. Based on this, we found that a sample size of 200 in each group would be sufficient to reject the null hypothesis if the true rate was 60%, which is why we estimated a total of 400-500 postvisit surveys in total would be needed to assess our outcomes. This sample size estimation was consistent with prior telephone interview studies at the University of Chicago with the same patient population and similar survey and interview techniques [7,53].

Adult GIM patients and pediatric parents who consented to the study were given the educational comic and a postvisit survey. Participants were instructed to (1) review the comic while waiting for their appointment and (2) complete the survey at the end of their visit. The postvisit survey included a question on whether participants would be interested in participating in a follow-up telephone interview at a mean of 8 months (range 3-12 months) after their clinic visit. Of those who expressed interest, 50 participants each from the adult and pediatric parent cohorts were selected at random for the interviews, which were conducted between July 2017 and October 2018. Participants orally consented to participate in the phone interview (Multimedia Appendix 2). A US $20 gift card was offered as compensation for their time. Phone interviews were digitally recorded and transcribed to ensure accuracy.

### Data Analysis

Descriptive statistics of patient postvisit surveys and phone interview responses were examined. Standard descriptive statistics were calculated including frequency counts and percentages, mean (standard deviation), or median. Univariate analyses were initially performed; since survey responses were on an ordinal Likert scale, nonparametric tests were used. Comparisons of survey responses involving three or more groups (eg, race) were made using Kruskal-Wallis tests, while comparisons involving two groups (eg, gender) were made using Wilcoxon rank sum tests. Associations between educational attainment and survey responses were examined using Spearman rank correlation coefficients. Pairwise comparisons were completed using Tukey’s honestly significant difference test. Phone survey versus postvisit survey response comparisons were completed using the Wilcoxon signed rank test for matched pairs. Multivariate analyses looking at whether gender, race, and education were independently associated with the odds of agreeing with a particular survey question (eg, “agree” was defined as a Likert response ≥4) were performed using logistic regression. Analysis was performed using Stata 14 (StataCorp LP, College Station, Texas). No adjustment for multiple testing was made. Our paper conforms to the SQUIRE 2.0 Revised Standards for Quality Improvement Reporting Excellence [54]. This study was approved by the University of Chicago’s Institutional Review Board.

## Results

### Overview

The study enrollment rate was 83.5% (197 consented/236 approached) for adult patients and 77.9% (325 consented/417 approached) for the pediatric parent cohort for a total of 522 participants (Table 2). In both cohorts, there were some patients who had at least one of the 18 survey questions missing an answer (142/197, 72.1% of adults and 104/325, 32% of pediatric parents). As such, data analyses are based on those who answered each question. In the adult cohort, the only significant difference in demographic characteristics between those who completed the entirety of the survey and those who did not was race distribution (*P*=.004), with 61% of noncompleters being African American compared to 46% of those who did complete it. In the pediatric cohort, the only statistically significant difference between survey completers and noncompleters was age, with noncompleters being significantly older (*P*<.001) than those who completed the survey.

The mean age was 58 (SD 17.3) years old for adult patients and 37 (SD 9.7) years old for pediatric parents. Overall, 65.6% (124/189) of adult patients and 85.8% (272/317) of pediatric parents were female, and 57.1% (104/182) of adult patients and 55.7% (176/316) of pediatric parents identified as African American. Less than half (72/181, 39.8%) of adult patients and a quarter (81/313, 25.9%) of pediatric parents reported educational attainment below a college degree, and 50.3% (91/181) of adult patients and 67.7% (212/313) of pediatric parents reported educational attainment at or above a bachelor’s degree. The average duration of the patient-physician relationship was 4.3 years in the GIM sample and 3.3 years in the pediatric sample.

**Table 2:** Participant demographics.

Participant demographics		Adult sample (n=197)	Pediatric parent sample (n=325)
**Age (years), n (%)**			
	18-19	2 (1.0)	5 (1.7)
	20-29	11 (5.8)	55 (18.2)
	30-39	19 (10.0)	138 (45.5)
	40-49	21 (11.1)	69 (22.8)
	50-59	40 (21.1)	29 (9.6)
	60 and older	97 (51.1)	7 (2.3)
**Gender, n (%)**			
	Female	124 (65.6)	272 (85.8)
	Male	65 (34.4)	45 (14.2)
**Race**			
	White	47 (25.8)	76 (24.1)
	African American	104 (57.1)	176 (55.7)
	Asian	17 (9.3)	18 (5.7)
	Hispanic or Latino	8 (4.4)	31 (9.8)
	Mixed/Other	6 (3.3)	15 (4.7)
**Educational attainment, n (%)**			
	Less than high school graduate	5 (2.8)	3 (1.0)
	High school graduate or GED^a^ equivalent	31 (17.1)	20 (6.4)
	Some college, no degree	36 (19.9)	58 (18.5)
	Associate degree	18 (9.9)	20 (6.4)
	Bachelor’s degree	31 (17.1)	81 (25.9)
	Graduate or professional degree	60 (33.2)	131 (41.9)
Length of relationship with physician (years), mean		4.3	3.3

^a^GED: General Educational Development.

### Postvisit Survey Results

#### Impact of Comic on Patient Advocacy to Enhance Patient-Physician-EHR Interactions

Nearly three-quarters of adult patients (115/162, 71.0%) and pediatric parents (224/313, 71.6%) agreed the comic “encouraged them to be more involved in the EHR.” Almost half of all participants (76/161, 47.2% of adult patients; 137/311, 44.1% of pediatric parents) agreed that the comic made them “feel more empowered about getting involved with the computer.” As a result of the comic, approximately a third of all participants (60/162, 37.0% of adult patients; 81/310, 26.1% of pediatric parents) asked to see the screen and to be more involved with their physician’s computer use by asking “to review their chart in EHR” (61/162, 37.7% of adult patients; 92/308, 29.9% of pediatric parents). As well, as a result of the comic, over one-third of participants (74/161, 46.0% of adult patients; 118/309, 38.2% of pediatric parents) felt more comfortable “asking their doctor to pay full attention to them if a sensitive topic came up.” More than half of participants (93/161, 57.8% of adult patients; 169/310, 54.5% of pediatric parents) felt that because of the comic, they were “more likely to get involved with their doctor’s computer use at future visits.” The remainder of the responses given on the entire survey are provided in Multimedia Appendix 3.

Based on univariate analyses, African American and Hispanic participants were more likely than White participants to “ask to see the screen” and “be involved due to the comic” (median 4 vs 3 for both; adult *P*=.01, *P*=.01; pediatric parent *P*=.02, *P*=.006, respectively). In both groups, lower educational attainment level was associated with significantly higher rates of self-reported advocacy behaviors to promote patient EHR engagement. Specifically, in the adult patient population, this included increased rates of “calling for physician attention” (ρ=−0.17, *P*=.04); and in the pediatric cohort, these behaviors included “asking to see the screen” (ρ=−0.18, *P*=.003) and “asking to be involved with the EHR” (ρ=−0.19, *P*<.001) as a result of the comic. Additionally, adult female patients were more likely than male patients to ask to be involved with their physician's computer use due to the comic (median 4 vs 3, *P*=.003); no gender differences were found in the pediatric parent population. Multivariate logistic regression analyses (Table 3) confirmed independent associations with education, especially in the pediatric cohort. In addition, robust associations with race and ethnicity remained in the pediatric cohort.

**Table 3:** Association between demographic characteristics and patient perceptions of comic in multivariate analyses.^a^

Characteristic				Statement “Because of the comic...”										
				I asked to see the screen		I asked to be more involved with the computer		I felt more empowered about getting involved with the computer		I felt more comfortable asking for the physician's full attention		I am more likely to get involved with the computer in the future		I think it's a good way to encourage involvement with the computer
**Adult cohort**														
	Female gender (vs male), odds ratio (95% CI)			1.01 (0.45-2.28)		0.78 (0.35-1.72)		1.49 (0.70-3.16)		0.96 (0.45-2.05)		1.55 (0.72-3.32)		1.65 (0.74-3.67)
	Education^b^, odds ratio (95% CI)			0.70** (0.54-0.90)		0.70** (0.54-0.90)		0.82 (0.64-1.04)		0.80 (0.63-1.01)		0.81 (0.64-1.03)		0.99 (0.77-1.28)
	**Race/ethnicity (vs white), odds ratio (95% CI)**													
		African American	2.00 (0.76-5.23)		1.69 (0.66-4.31)		1.05 (0.45-2.48)		1.12 (0.47-2.64)		1.49 (0.63-3.51)		1.52 (0.61-3.76)	
		Asian	2.21 (0.54-8.97)		1.26 (0.30-5.27)		1.08 (0.30-3.88)		1.24 (0.34-4.46)		2.72 (0.72-10.19)		1.11 (0.30-4.11)	
		Hispanic	3.49 (0.59-20.52)		2.81 (0.49-16.22)		1.50 (0.28-8.22)		3.18 (0.52-19.53)		2.90 (0.47-18.06)		3.70 (0.39-35.42)	
		Other	N/A^c^		N/A		N/A		0.50 (0.04-5.55)		1.05 (0.13-8.70)		1.37 (0.13-14.79)	
	Model chi-square (*df*)			15.6 (5)		14.2 (5)		5.2 (5)		7.2 (6)		8.4 (6)		3.8 (6)
	*P* value			0.008		0.01		0.39		0.31		0.21		0.71
	n			135		135		135		138		138		140
**Pediatric cohort**														
	Female gender (vs male), odds ratio (95% CI)			0.86 (0.39-1.91)		0.95 (0.43-2.10)		1.13 (0.56-2.31)		1.32 (0.64-2.75)		0.64 (0.32-1.30)		0.97 (0.46-2.06)
	Education^b^, odds ratio (95% CI)			0.75** (0.61-0.91)		0.74** (0.61-0.90)		0.76** (0.63-0.92)		0.72*** (0.59-0.87)		0.78* (0.65-0.95)		0.84 (0.68-1.05)
	**Race/ethnicity (vs white), odds ratio (95% CI)**													
		African American	2.41 (0.99-5.87)		3.68** (1.45-9.34)		1.34 (0.71-2.54)		0.93 (0.48-1.80)		1.41 (0.76-2.61)		1.41 (0.73-2.72)	
		Asian	3.64 (0.97-13.59)		5.75** (1.54-21.42)		2.04 (0.68-6.12)		2.81 (0.93-8.51)		1.08 (0.37-3.17)		0.72 (0.24-2.15)	
		Hispanic	5.03** (1.66-15.20)		6.17** (1.95-19.56)		1.72 (0.68-4.35)		1.25 (0.48-3.21)		1.29 (0.51-3.27)		0.87 (0.33-2.30)	
		Other	4.54* (1.14-18.06)		12.60*** (3.00-52.98)		7.28** (1.82-29.11)		2.67 (0.80-8.87)		4.11* (1.04-16.32)		2.75 (0.56-13.46)	
	Model chi-square (*df*)			23.9 (6)		34.3 (6)		20.3 (6)		18.6 (6)		15.1 (6)		8.2 (6)
	*P* value			<.001		<.001		0.003		0.005		0.02		0.22
	n			290		287		290		288		288		291

^a^Numbers in table are odds ratios (95% CI) from 6 separate multivariate logistic regression models for agreeing with given statement (agree or strongly agree vs not). * *P*<.05, ** *P*<.01, *** *P*<.001.

^b^Treated as a continuous measure using integer scores for educational level (higher scores =  more education).

^c^Not applicable.

#### Satisfaction With Physician EHR Use

The large majority of adult patients (151/192, 78.6%) and pediatric parents (294/323, 91.0%) agreed that their physician “made sure they could see the screen” during the clinic visit and “made sure they could talk face to face even though they were using the computer” (180/194, 92.8% and 301/325, 92.6%, respectively). Most adult patients (128/189, 67.7%) and pediatric parents (260/325, 80%) agreed that their physician encouraged them to “interact with the computer” (eg, showing information in EHR, encouraging them to use the patient portal). Nearly three-quarters of adult patients (125/172, 72.7%) and pediatric parents (247/325, 76%) agreed their “physician valued the computer and was positive about the benefits.”

#### Perceptions of Physician Communication at Current Visit Compared to Prior Visits

When comparing the current visit with recollections of prior visits with the same physician, more than half of participants (109/163, 66.9% of adult patients; 186/325, 57.2% of pediatric parents) agreed that at the current visit, their physician “used the computer more effectively to communicate with them” and was “less distracted by the computer and more focused on them” (97/157, 61.8% of patients; 186/325, 57.2% of pediatric parents). Further, compared to prior visits, more than half of all participants (99/160, 61.9% of adult patients; 179/325, 55.1% of pediatric parents) agreed that they “understood more about their/their child’s health and plan,” and 56.2% (81/144) of adult patients and 46.6% (131/281) of pediatric parents were “more satisfied with their relationship with their/their child’s doctor because of how they used the computer with them.”

### Follow-up Telephone Interview

A total of 148 adult patients (148/197, 75.1%) and 196 pediatric parents (196/325, 60.3%) were interested in participating in follow-up phone interviews. Patients were randomly selected from this group, and a total of 83 adult patients (83/148, 56.1%) and 60 pediatric parents (60/196, 30.6%) were called to reach 50 completed interviews for each cohort. Follow-up phone interviews were conducted on average 8 months (range 3-12 months) post visit. There were no significant differences in age, sex, race, educational attainment level, or length of physician relationship between those that completed phone interviews, those that were interested in taking part in phone interviews but did not (eg, they were unavailable or were not randomly selected to take part), and those that were only initially surveyed after their visit and were not interviewed by phone because they declined to take part.

All pediatric parents (50/50) and 90% (45/50) of adult patients remembered the comic, and almost half of adult patients (23/50, 46%) and pediatric parents (21/50, 42%) recalled at least one of the comic’s three ABC best-practice behaviors without prompting. When asked if they used the advocacy behaviors suggested in the comic at subsequent physician visits, adult patients reported that they were more likely to ask to see the screen (Multimedia Appendix 2, question 3, median response 3 vs 4, *P*=.006), and pediatric parents reported increases in asking to see the screen (Multimedia Appendix 2, question 3, median response 3 vs 4, *P*<.001) and calling for physician attention (Multimedia Appendix 2, question 4, median response 3 vs 4, *P*<.001). Pediatric parents also felt that the comic had encouraged them to speak up and get more involved with physician computer use since the index visit (Multimedia Appendix 2, question 2, median response 4 vs 4, *P*=.02) and that it made them feel more empowered to get involved with computer use at future visits (Multimedia Appendix 2, question 5, median response 3 vs 4, *P*<.001). There were no significant differences in adults feeling more empowered to get involved at future visits (Multimedia Appendix 2, question 5, median response 4 vs 4, *P*=0.23) or in either group thinking the comic was effective in encouraging continued involvement with the computer at physician visits (Multimedia Appendix 2, question 6, median response 5 vs 4, *P*=.26 for adults; median response 4 vs 4, *P*=.06 for pediatrics).

Open-ended question responses were collectively pooled. Content analysis identified unique themes, subthemes, and representative quotations in order to build a picture of the respondents’ collective experiences (Table 4) [55].

**Table 4:** Themes and subthemes relevant to the educational comic and EHR use.

Themes and subthemes			Representative quotes
**Patient perceptions**			
	EHR^a^ awareness	“The effort as a whole did make me more aware of the computer and I feel like, oh, I notice the screen and the doctor’s use”	
	Screen viewing	“The comic was great because I didn’t know it was my right to look at the computer”	
	Asking questions	“The comic was really good; I wasn’t sure if you could ask questions”	
	Time for EHR involvement	“Patients often feel like they are rushed, the comic gives assurance that its okay to ask questions”	
	Encouraging engagement	“I already do the ABCs; for someone who is more bashful or reserved, the comic may be more helpful.”	
**Patient behaviors**			
	EHR engagement	“I've had several appointments since the appointment and it's been much better, I was very involved, one physician did on a laptop which was cool so I could see.”	
	Asked to see screen	“Comic was first time to see the screen. Comic helped me ask, prior to the visit I had never asked to see the screen”	
	Asked for clarification	“I ask can I see the screen, talk to me about what you see”	
	Asked about clinician behaviors	“Asked him to further explain to me what he was doing and inputting on the computer”	
	Corrected errors	“Asked to see my record and make corrections”	
	Watched what clinician was typing	“I liked to see what she is typing. Also it helps me understand what is happening during our visit. Great idea.”	
	Asked to see things in the EHR	“When showing child growth, I asked to see the graph”	
**Physician behaviors**			
	EHR use in visit	“My doctor is awesome, when she's pulling up my history and my labs she pulls up the screen so I can see it and she looks at my medications and she asks me are you taking this, are you still taking them twice a day”	
	Patient portal use	“My doctor involved me by encouraging me to go online and look at the chart”	
**Suggestions for comic modification**			
	Improved readability	“Bigger font in speech bubbles, more lay language”	
	Translation	“Have it in other languages such as Spanish”	
	Increased visibility	“Place cartoon in rooms, on the wall”	
	Provide script examples	“Like using key phrases / trigger points, give phrases that patients can use”	
	Orientation to EHR content	“Give more examples of what one may find on computer screen that he/she may wish to see”	
	Highlight benefits of involvement	“Give more detailed examples of the benefits of getting involved”	
	Highlight drawbacks of uninvolvement	“I would add an example that would scare them to get involved”	
**Suggestions for EHR engagement**			
	Patient-facing portions of EHR	“Have a portion of the EHR where pts can interact w/computer themselves”	
	Mobile technology	“A tablet to follow along with the chart as doc is on computer”	
	Patient portal training	“If someone showed me how to use MyChart”	
	General technology training	“Teaching us how to use a computer and how to learn”	
	Room ergonomics	“Screen where patient and doc can see without doc’s back to patient”	
	Nursing involvement	“Nurses can tell patients/parents to ask dr to share computer screen”	
	Highlight importance of patient involvement	“Maybe take a moment at the beginning to reiterate what they’re doing every step of the way on the computer and let patients know that they have the right to see the screen - gives partnership in their own personal care”	
	Physician training	“Train the doctor to be move involved”	
	Reset physician EHR expectations	“Wish drs had to always show info unless confidential info is on screen”	

^a^EHR: electronic health record.

## Discussion

To our knowledge, this is the first study evaluating the impact of an educational comic on patient advocacy for enhanced patient-physician-EHR interactions. This easily replicable intervention may help improve patient self-advocacy for patient-centered engagement with the EHR in pediatric and adult primary care settings, which can promote both patient education and satisfaction with physician EHR use. Importantly, the effect was more pronounced in African American and Hispanic patients and patients with lower levels of educational attainment.

Prior studies have found that non-White patients, those with lower educational attainment, and non–English-speaking patients experience health care disparities which may result in poor health outcomes [35-47]. These patients may also come to visits with lower levels of health literacy and agency, which can be associated with difficulty understanding their diagnoses and treatment plans [35-47]. The open-ended comments in our study (Table 4) highlighted that some patients do not feel empowered to ask questions during their visits, and handing out the educational comic may serve as a simple but powerful invitation to speak up and ask questions of their physicians.

Additionally, patients from disadvantaged backgrounds are more likely to report distrust of their health care team when compared to patients from nondisadvantaged social and educational backgrounds [44,45,47,56]. Sharing the EHR screen and enhancing transparency and engagement with the EHR may increase a patient’s sense of partnership and trust with their physician, which may help promote increased trust of the medical system [26,44-47]. Moreover, patients from disadvantaged groups may need more formal encouragement to engage with their physicians and the EHR, which is important because enhanced engagement with providers and health care technology can help increase patient understanding of care plans and improve preparation for future visits [6,12,44-47,57]. Our educational comic may be used as a tool to empower vulnerable patients to be more engaged in their care and promote agency. In addition, patients with limited health literacy may rely on health information from social media and blogs, which can contain lower quality health information [47]. Encouraging patients to ask their physicians questions may help dispel health myths, promote health literacy, and help reduce health disparities [44-47].

With regard to patient satisfaction with physician EHR use, patients reported that their physicians demonstrated more patient-centered behaviors when using the EHR at the index visit as compared to prior visits. This may be due to the patient’s increased EHR engagement during the visit, which could have prompted physicians to engage in more patient-centered EHR behaviors. Future research is needed to better understand how enhanced patient EHR engagement is perceived by physicians and the impact on physicians’ EHR-related behavior.

Lastly, there were no significant differences in either adult or pediatric respondents thinking the comic was effective at encouraging continued involvement with the computer on phone follow-up. However, what is perhaps more important is that when describing the comic’s impacts on specific behaviors at subsequent physician visits, both adult and pediatric patients reported increased use of the self-advocacy behaviors in the comic since their initial visit, particularly in the pediatric cohort; this perhaps suggests that it may have been effective in contributing to lasting impacts on their subsequent EHR interactions, especially when advocating on behalf of someone else (ie, their child).

Our study has several limitations. First, it was a single-institution study, and we had an overrepresentation of women and advanced degree holders in our sample, both of which may limit generalizability. In addition, while it may be difficult to directly compare pediatric parent to adult patient responses, findings from pediatric parents may be generalizable to family members who accompany adult patients to visits or serve as proxies for those who cannot speak or advocate for themselves. Our study did not include a control group, and we did not conduct a preintervention survey due to resource constraints. To adjust for this, the postvisit survey asked participants to rate their perceptions, advocacy behaviors, and satisfaction with their physician’s EHR use at the current visit as compared to their recollection of these measures from prior visits with the same providers. These responses may have been subject to recall and response bias, and phone interviews may have been affected by the variable follow-up period. Our findings were dependent on reports from adult patients and pediatric parent participants without direct observation of physician or patient behavior. Further, we did not include a control group with a text-only nongraphic version of the comic, so it is not possible to say if a nongraphic intervention would have had the same impacts. Lastly, physicians were generally deemed by their patients to be adept at engaging them with the EHR, perhaps because they were biased to providing positive responses, and physicians were aware that the study was occurring, which may have influenced their EHR behaviors. In order to help minimize this impact, physicians were not shown the patient comic or the survey. 

Further work is needed to understand how to tailor educational comics to different patient populations and clinical settings, such as the inpatient hospital environment, to effectively engage patients and physicians with the EHR. While this educational intervention targeted patients, it is also important to teach patient-centered EHR behaviors to physicians to promote patient-physician-EHR engagement [20-26,58,59], and these efforts should be pursued in tandem. Additionally, EHRs should evolve to account for user experience, patient health literacy levels, and language needs to help reduce the digital divide and health disparities [19,60-68].

In conclusion, to our knowledge, this is the first study evaluating the impact of an educational comic intervention on patient-centered EHR use and patient self-advocacy for EHR engagement. We found that our educational comic was well received, participant ratings showed benefits in the outcomes measured, and there was no harm to participants as a result of their participation. Our comic may be effective in promoting patient-driven initiatives to enrich patient-physician-EHR interactions and may be most impactful in engaging African American patients, Hispanic patients, and patients with lower educational attainment. This simple intervention can be easily replicated, and future work should focus on studying the impact of the educational comic in other clinical settings and objectively measuring behaviors related to the patient-physician-EHR interaction. Educational comics should be considered in future initiatives to promote patient education and humanistic patient-centered EHR use.

## Appendix

Multimedia Appendix 1 Adult patient comic initial survey.

Multimedia Appendix 2 Adult patient phone interview script.

Multimedia Appendix 3 Patient perceptions of comic intervention: postvisit survey results.

## Abbreviations

EHR: electronic health record
GIM: general internal medicine
PPC: pediatric primary care
SQUIRE: Standards for Quality Improvement Reporting Excellence

## References

[1] Colicchio TK, Cimino JJ, Del Fiol G (2019). Unintended Consequences of Nationwide Electronic Health Record Adoption: Challenges and Opportunities in the Post-Meaningful Use Era. J Med Internet Res.

[2] Percentage of office-based physicians using any electronic health record (EHR)/electronic medical record (EMR) system and physicians that have a certified EHR/EMR system, by U.S. state: National Electronic Health Records Survey, 2017.

[3] Office-based Physician Electronic Health Record Adoption - Health IT Dashboard.

[4] Crampton NH, Reis S, Shachak A (2016). Computers in the clinical encounter: a scoping review and thematic analysis. J Am Med Inform Assoc.

[5] Shachak A, Reis S (2009). The impact of electronic medical records on patient-doctor communication during consultation: a narrative literature review. J Eval Clin Pract.

[6] Alkureishi MA, Lee WW, Lyons M, Press VG, Imam S, Nkansah-Amankra A, Werner D, Arora VM (2016). Impact of Electronic Medical Record Use on the Patient-Doctor Relationship and Communication: A Systematic Review. J Gen Intern Med.

[7] Lee WW, Alkureishi MA, Ukabiala O, Venable LR, Ngooi SS, Staisiunas DD, Wroblewski KE, Arora VM (2016). Patient Perceptions of Electronic Medical Record Use by Faculty and Resident Physicians: A Mixed Methods Study. J Gen Intern Med.

[8] Kazmi Z (2013). Effects of exam room EHR use on doctor-patient communication: a systematic literature review. Inform Prim Care.

[9] Rathert C, Mittler JN, Banerjee S, McDaniel J (2017). Patient-centered communication in the era of electronic health records: What does the evidence say?. Patient Educ Couns.

[10] Khoong EC, Cherian R, Matta GY, Lyles CR, Schillinger D, Ratanawongsa N (2019). Perspectives of English, Chinese, and Spanish-Speaking Safety-Net Patients on Clinician Computer Use: Qualitative Analysis. J Med Internet Res.

[11] Antoun J, Hamadeh G, Romani M (2019). Effect of computer use on physician-patient communication using interviews: A patient perspective. Int J Med Inform.

[12] Wolfe L, Chisolm MS, Bohsali F (2018). Clinically Excellent Use of the Electronic Health Record: Review. JMIR Hum Factors.

[13] Shaarani I, Taleb R, Antoun J (2017). Effect of computer use on physician-patient communication using a validated instrument: Patient perspective. Int J Med Inform.

[14] Rathert C, Wyrwich MD, Boren SA (2013). Patient-centered care and outcomes: a systematic review of the literature. Med Care Res Rev.

[15] Gluyas H (2015). Patient-centred care: improving healthcare outcomes. Nurs Stand.

[16] Robinson JH, Callister LC, Berry JA, Dearing KA (2008). Patient-centered care and adherence: definitions and applications to improve outcomes. J Am Acad Nurse Pract.

[17] Wildevuur SE, Simonse LWL (2015). Information and communication technology-enabled person-centered care for the "big five" chronic conditions: scoping review. J Med Internet Res.

[18] Finkelstein J, Knight A, Marinopoulos S, Gibbons MC, Berger Z, Aboumatar H, Wilson RF, Lau BD, Sharma R, Bass EB (2012). Enabling patient-centered care through health information technology. Evid Rep Technol Assess (Full Rep).

[19] Yang Y, Asan O (2016). Designing Patient-facing Health Information Technologies for the Outpatient Settings: A Literature Review. J Innov Health Inform.

[20] Alkureishi MA, Lee WW, Webb S, Arora V (2018). Integrating Patient-Centered Electronic Health Record Communication Training into Resident Onboarding: Curriculum Development and Post-Implementation Survey Among Housestaff. JMIR Med Educ.

[21] Lee WW, Alkureishi ML, Isaacson JH, Mayer M, Frankel RM, London DA, Wroblewski KE, Arora VM (2018). Impact of a brief faculty training to improve patient-centered communication while using electronic health records. Patient Educ Couns.

[22] Lee WW, Alkureishi ML, Wroblewski KE, Farnan JM, Arora VM (2017). Incorporating the human touch: piloting a curriculum for patient-centered electronic health record use. Med Educ Online.

[23] Alkureishi MA, Lee WW, Lyons M, Wroblewski K, Farnan JM, Arora VM (2018). Electronic-clinical evaluation exercise (e-CEX): A new patient-centered EHR use tool. Patient Educ Couns.

[24] Shachak A, Randhawa GK, Crampton NH (2019). Educational approaches for improving physicians' use of health information technology. Healthc Manage Forum.

[25] Patel MR, Smith A, Leo H, Hao W, Zheng K (2019). Improving Patient-Provider Communication and Therapeutic Practice Through Better Integration of Electronic Health Records in the Exam Room: A Pilot Study. Health Educ Behav.

[26] Duke P, Frankel RM, Reis S (2013). How to integrate the electronic health record and patient-centered communication into the medical visit: a skills-based approach. Teach Learn Med.

[27] Arya R, Ichikawa T, Callender B, Schultz O, DePablo M, Novak K, Li S, Shenoy A, Everman A, Braunstein S, Dec I, Lala S, Feng Y, Biltz L, McCall AR, Golden DW (2020). Communicating the External Beam Radiation Experience (CEBRE): Perceived Benefits of a Graphic Narrative Patient Education Tool. Pract Radiat Oncol.

[28] Furuno Y, Sasajima H (2015). Medical Comics as Tools to Aid in Obtaining Informed Consent for Stroke Care. Medicine (Baltimore).

[29] Rosas-Blum ED, Granados HM, Mills BW, Leiner M (2018). Comics as a Medium for Parent Health Education: Improving Understanding of Normal 9-Month-Old Developmental Milestones. Front Pediatr.

[30] Sridhar A, Friedman S, Grotts JF, Michael B (2019). Effect of theory-based contraception comics on subjective contraceptive knowledge: a pilot study. Contraception.

[31] Mendelson A, Rabinowicz N, Reis Y, Amarilyo G, Harel L, Hashkes PJ, Uziel Y (2017). Comics as an educational tool for children with juvenile idiopathic arthritis. Pediatr Rheumatol Online J.

[32] Pieper C, Homobono A (2000). Comic as an education method for diabetic patients and general population. Diabetes Research and Clinical Practice.

[33] Green MJ, Myers KR (2010). Graphic medicine: use of comics in medical education and patient care. BMJ.

[34] Ashwal G, Thomas A (2018). Are Comic Books Appropriate Health Education Formats to Offer Adult Patients?. AMA J Ethics.

[35] Vanderbilt AA, Isringhausen KT, VanderWielen LM, Wright MS, Slashcheva LD, Madden MA (2013). Health disparities among highly vulnerable populations in the United States: a call to action for medical and oral health care. Med Educ Online.

[36] Piccardi C, Detollenaere J, Vanden Bussche P, Willems S (2018). Social disparities in patient safety in primary care: a systematic review. Int J Equity Health.

[37] Vollbrecht H, Arora V, Otero S, Carey K, Meltzer D, Press VG (2020). Evaluating the Need to Address Digital Literacy Among Hospitalized Patients: Cross-Sectional Observational Study. J Med Internet Res.

[38] Cruz-Flores S, Rabinstein A, Biller J, Elkind MSV, Griffith P, Gorelick PB, Howard G, Leira EC, Morgenstern LB, Ovbiagele B, Peterson E, Rosamond W, Trimble B, Valderrama AL, American Heart Association Stroke Council, Council on Cardiovascular Nursing, Council on EpidemiologyPrevention, Council on Quality of CareOutcomes Research (2011). Racial-ethnic disparities in stroke care: the American experience: a statement for healthcare professionals from the American Heart Association/American Stroke Association. Stroke.

[39] Flores G, Committee On Pediatric Research (2010). Technical report--racial and ethnic disparities in the health and health care of children. Pediatrics.

[40] Berkman ND, Sheridan SL, Donahue KE, Halpern DJ, Crotty K (2011). Low health literacy and health outcomes: an updated systematic review. Ann Intern Med.

[41] Cutilli CC, Simko LC, Colbert AM, Bennett IM (2018). Health Literacy, Health Disparities, and Sources of Health Information in U.S. Older Adults. Orthop Nurs.

[42] Mouton CP, Hayden M, Southerland JH (2017). Cardiovascular Health Disparities in Underserved Populations. Prim Care.

[43] Sklar DP (2017). New Conversations: Justice, Disparities, and Meeting the Needs of Our Most Vulnerable Populations. Acad Med.

[44] Walker J, Leveille S, Bell S, Chimowitz H, Dong Z, Elmore JG, Fernandez L, Fossa A, Gerard M, Fitzgerald P, Harcourt K, Jackson S, Payne TH, Perez J, Shucard H, Stametz R, DesRoches C, Delbanco T (2019). OpenNotes After 7 Years: Patient Experiences With Ongoing Access to Their Clinicians' Outpatient Visit Notes. J Med Internet Res.

[45] Gerard M, Chimowitz H, Fossa A, Bourgeois F, Fernandez L, Bell SK (2018). The Importance of Visit Notes on Patient Portals for Engaging Less Educated or Nonwhite Patients: Survey Study. J Med Internet Res.

[46] Delbanco T, Walker J, Bell SK, Darer JD, Elmore JG, Farag N, Feldman HJ, Mejilla R, Ngo L, Ralston JD, Ross SE, Trivedi N, Vodicka E, Leveille SG (2012). Inviting patients to read their doctors' notes: a quasi-experimental study and a look ahead. Ann Intern Med.

[47] Chen X, Hay JL, Waters EA, Kiviniemi MT, Biddle C, Schofield E, Li Y, Kaphingst K, Orom H (2018). Health Literacy and Use and Trust in Health Information. J Health Commun.

[48] Elsabrout K (2018). Increasing diabetic patient engagement and self-reported medication adherence using a web-based multimedia program. J Am Assoc Nurse Pract.

[49] Unk JA, Brasington R (2014). Efficacy study of multimedia rheumatoid arthritis patient education program. J Am Assoc Nurse Pract.

[50] Jonikas JA, Grey DD, Copeland ME, Razzano LA, Hamilton MM, Floyd CB, Hudson WB, Cook JA (2013). Improving propensity for patient self-advocacy through wellness recovery action planning: results of a randomized controlled trial. Community Ment Health J.

[51] Dryden EM, Desmarais J, Arsenault L (2014). Effectiveness of the IMPACT:Ability program to improve safety and self-advocacy skills in high school students with disabilities. J Sch Health.

[52] Hagan TL, Cohen S, Stone C, Donovan H (2016). Theoretical to Tangible: Creating a Measure of Self-Advocacy for Female Cancer Survivors. J Nurs Meas.

[53] Pincavage AT, Lee WW, Beiting KJ, Arora VM (2013). What do patients think about year-end resident continuity clinic handoffs? A qualitative study. J Gen Intern Med.

[54] Ogrinc G, Davies L, Goodman D, Batalden P, Davidoff F, Stevens D (2016). SQUIRE 2.0 (Standards for QUality Improvement Reporting Excellence): revised publication guidelines from a detailed consensus process. BMJ Qual Saf.

[55] Braun V, Clarke V (2006). Using thematic analysis in psychology. Qualitative Research in Psychology.

[56] Street RL, O'Malley KJ, Cooper LA, Haidet P (2008). Understanding concordance in patient-physician relationships: personal and ethnic dimensions of shared identity. Ann Fam Med.

[57] Asan O, Tyszka J, Fletcher KE (2016). Capturing the patients' voices: Planning for patient-centered electronic health record use. Int J Med Inform.

[58] Lanier C, Dominicé Dao M, Hudelson P, Cerutti B, Junod Perron N (2017). Learning to use electronic health records: can we stay patient-centered? A pre-post intervention study with family medicine residents. BMC Fam Pract.

[59] Graham-Jones P, Jain SH, Friedman CP, Marcotte L, Blumenthal D (2012). The need to incorporate health information technology into physicians' education and professional development. Health Aff (Millwood).

[60] Carayon P, Hoonakker P (2019). Human Factors and Usability for Health Information Technology: Old and New Challenges. Yearb Med Inform.

[61] Schickedanz A, Huang D, Lopez A, Cheung E, Lyles CR, Bodenheimer T, Sarkar U (2013). Access, interest, and attitudes toward electronic communication for health care among patients in the medical safety net. J Gen Intern Med.

[62] Lyles CR, Tieu L, Sarkar U, Kiyoi S, Sadasivaiah S, Hoskote M, Ratanawongsa N, Schillinger D (2019). A Randomized Trial to Train Vulnerable Primary Care Patients to Use a Patient Portal. J Am Board Fam Med.

[63] Gibbons MC, Casale CR (2010). Reducing disparities in health care quality: the role of health IT in underresourced settings. Med Care Res Rev.

[64] Sorondo B, Allen A, Bayleran J, Doore S, Fathima S, Sabbagh I, Newcomb L (2016). Using a Patient Portal to Transmit Patient Reported Health Information into the Electronic Record: Workflow Implications and User Experience. EGEMS (Wash DC).

[65] Beacom AM, Newman SJ (2010). Communicating health information to disadvantaged populations. Fam Community Health.

[66] Czaja SJ, Zarcadoolas C, Vaughon WL, Lee CC, Rockoff ML, Levy J (2015). The usability of electronic personal health record systems for an underserved adult population. Hum Factors.

[67] Tieu L, Sarkar U, Schillinger D, Ralston JD, Ratanawongsa N, Pasick R, Lyles CR (2015). Barriers and Facilitators to Online Portal Use Among Patients and Caregivers in a Safety Net Health Care System: A Qualitative Study. J Med Internet Res.

[68] Tieu L, Schillinger D, Sarkar U, Hoskote M, Hahn KJ, Ratanawongsa N, Ralston JD, Lyles CR (2016). Online patient websites for electronic health record access among vulnerable populations: portals to nowhere?. J Am Med Inform Assoc.

