# Supplementary material for: Impact of an Educational Comic to Enhance Patient-Physician–Electronic Health Record Engagement: Prospective Observational Study
Source: JMIR Hum Factors. 2021 Apr 28;8(2):e25054. doi: 10.2196/25054 (PMC8116991; doi:10.2196/25054)
Supplement: Multimedia Appendix 1 [file humanfactors_v8i2e25054_app1.docx]

## Appendix 1:

**Adult Patient Comic Initial Survey**

**1. Name**: _____________________________2. **Age**: _____ 3. **Sex**: ❒ Male ❒ Female

**4. Education**: What is the **HIGHEST** degree or level of school you completed*?*

❒Less than high school ❒High school graduate (includes equivalency)

❒Some college, no degree ❒Associate's degree

❒Bachelor's degree. ❒Graduate or professional degree

**5. Race**: ❒White ❒ African American ❒Asian ❒Hispanic ❒Pacific Islander ❒Other _________

**6. How long have you been seeing your doctor? ______________________________________**

**7. Name of doctor you saw today: _________________________________________________**

| **During your clinic visit TODAY,  the doctor** | **Strongly Disagree** | **Disagree** | **Neutral** | **Agree** | **Strongly**  **Agree** | **Unsure** |
| --- | --- | --- | --- | --- | --- | --- |
| 1. … made sure the computer was positioned so I could **see the screen**& what they were doing. | 1 | 2 | 3 | 4 | 5 | U |
| 1. …made sure I could talk **face to face** even though they were using the computer (e.g. the doctor did not have his back to you) | 1 | 2 | 3 | 4 | 5 | U |
| 1. …encouraged me to **interact** with the computer like showing us information in my **chart** (lab results, images), sharing **websites** or **handouts** on the computer, encouraging me to sign up for **MyChart** online. | 1 | 2 | 3 | 4 | 5 | U |
| 1. …knew if it **was not** a good time to use the computer like when a **sensitive topic** came up they **stopped using the computer** & paid full attention to me. | 1 | 2 | 3 | 4 | 5 | U |
| 1. …used the computer to **educate** me about our child’s health, medical conditions or treatment. | 1 | 2 | 3 | 4 | 5 | U |
| 1. ….**valued the computer** and was positive about the benefits of using the computer. | 1 | 2 | 3 | 4 | 5 | U |
| **COMPARED TO YOUR LAST VISIT with the same doctor…** | **Strongly Disagree** | **Disagree** | **Neutral** | **Agree** | **Strongly**  **Agree** | **Unsure** |
| 1. Today, my doctor used the **computer** to **more effectively communicate** with me. | 1 | 2 | 3 | 4 | 5 | U |
| 1. Today, my doctor was **less distracted** by the computer and more focused on me. | 1 | 2 | 3 | 4 | 5 | U |
| 1. Today, I **understood more** about my health or plan because of how my doctor **used the computer** to provide education | 1 | 2 | 3 | 4 | 5 | U |
| 1. Today, my doctor made a better effort to **involve me with the computer** | 1 | 2 | 3 | 4 | 5 | U |
| 1. Today, my doctor was better about **sharing the screen with me** and allowing me to follow along | 1 | 2 | 3 | 4 | 5 | U |
| 1. Today, I was **more satisfied with our RELATIONSHIP** with my doctor because of how they **used the computer**. | 1 | 2 | 3 | 4 | 5 | U |
| **Regarding the Comic you received today at check-in …** | **Strongly Disagree** | **Disagree** | **Neutral** | **Agree** | **Strongly**  **Agree** | U |
| 1. Because of the **COMIC, I ASKED to see the screen** and what my doctor was doing with the computer. | 1 | 2 | 3 | 4 | 5 | U |
| 1. Because of the **COMIC, I ASKED TO BE MORE INVOLVED with the computer** like asking to review my chart in the computer & asking questions about what I saw | 1 | 2 | 3 | 4 | 5 | U |
| 1. Because of the **COMIC, I felt more EMPOWERED** about **getting involved** with the computer. | 1 | 2 | 3 | 4 | 5 | U |
| 1. Because of the COMIC, I felt more comfortable about **ASKING the doctor to pay full attention to me if a sensitive topic** came up. | 1 | 2 | 3 | 4 | 5 | U |
| 1. Because of the **COMIC, in the FUTURE, I WILL BE MORE LIKELY to** get involved with the computer at **my** visits. | 1 | 2 | 3 | 4 | 5 | U |
| 1. The **COMIC** is a **good way to encourage** people to get involved with the computer at doctor visits. | 1 | 2 | 3 | 4 | 5 | U |

26. **Did your doctor involve you with the COMPUTER?** ❒ YES ❒ NO

27. **Were there POSITIVE ways your doctor involved YOU with the COMPUTER?**❒ NO ❒ YES – If yes, LIST EXAMPLES HERE: ________________________________________________

_____________________________________________________________________________________

28. **Were there NEGATIVE ways your doctor involved YOU with the COMPUTER?**❒ NO ❒ YES – If yes, LIST EXAMPLES HERE: ________________________________________________
_____________________________________________________________________________________

29. **How would you CHANGE THE COMIC to BETTER encourage people to get involved with the computer?** ❒ NO ❒ YES – If yes, LIST EXAMPLES HERE: ______________________________________

_____________________________________________________________________________________

30. **Are there OTHER WAYS we can ENCOURAGE patients to get involved with the computer?**❒ NO ❒ YES – If yes, LIST EXAMPLES HERE: ________________________________________________ _____________________________________________________________________________________

31. **Do you have comments about how your doctor’s use of the computer affects your RELATIONSHIP with the doctor or your ability to communicate with him/her?**

❒ NO ❒ YES – If yes, LIST EXAMPLES HERE: ________________________________________________

_____________________________________________________________________________________

32.**Do you wish to participate in a SHORT follow-up phone survey?** You will be compensated with a **$20 gift card to your choice of Amazon, Domino’s Pizza, Wendy’s or Quiznos.**

❒ NO ❒ YES – If yes, phone number: _____________________________________________________

33. Best times to call (check all that apply): 🞎Mornings (9-12) 🞎Afternoons (12-5pm) 🞎Evenings (5-7pm)
