# Supplementary material for: Impact of an Educational Comic to Enhance Patient-Physician–Electronic Health Record Engagement: Prospective Observational Study
Source: JMIR Hum Factors. 2021 Apr 28;8(2):e25054. doi: 10.2196/25054 (PMC8116991; doi:10.2196/25054)
Supplement: Multimedia Appendix 2 [file humanfactors_v8i2e25054_app2.docx]

## Appendix 2:

**Adult Patient Phone Interview Script**

**I’d like to repeat myself in order to get this on tape: Do I have your permission to record this call?... ********* Identify interviewee, state the date.**************

Do you remember the comic we gave you at your doctor’s visit in xxx? The comic

was about how to get more involved during your clinic visit when your doctor is using the computer.

(The patient will answer yes or no. If yes, the interviewer will proceed to the script below. If no the interviewer will say the following script and skip to question 8: The comic summarized three tips (the ABCs) to get more involved with our doctor’s computer use during the clinic visit: A is for Ask to see the screen, B is to ask to Be involved in with computer use, and C is for Call for attention and ask for your doctor’s full when a sensitive or important topic comes up)

I am going to ask you some follow-up questions about the comic and get your opinion on ways to improve communication between patients and doctors when a computer is used.

1. Do you remember any of the three tips, the ABCs, addressed on the comic? Yes or No

- If Yes, what were they?
- If No, interviewer will remind them of three tips: Ask to see the screen, ask to Be involved in with computer use, Call for attention and ask for your doctor’s full when a sensitive or important topic comes up

**On a scale of strongly disagree to strongly agree,** where 1 is strongly disagree 2 is Disagree 3 is neutral , 4 is agree and 5 is strongly agree, how would you rate these statements?

Since your visit to your doctor in xxx…

2. The comic encouraged me to speak up and **get more involved** with the computer at **my** subsequent visits with my doctor.

3. The comic encouraged me to **ask to see the screen at subsequent doctor’s appointments** to allow me to follow along on the computer.

4. The comic made me feel more comfortable about **ASKING doctors to pay full attention to me if a sensitive topic** came up.

5**.** The comic made me feel more **EMPOWERED** **about asking to get more involved** with the computer at future doctor’s visits.

6. The **COMIC** was effective in continuing to **encourage** me to get involved with the computer at doctor visits.

These next few questions are open ended:

7. Do you have suggestions for how we can **improve the comic** to make patients feel more empowered to get involved with the computer at visits?

8. Besides the comic, **what else can we do** to help patients feel more empowered to get involved with the computer at their visits?

9. Can you give me some examples of **how you’ve asked to get more involved with your doctors use of the computer during your clinic visits**?

10. In the future would you be open to us contacting you again over the phone if we have further questions about this issue?

That is the end of our survey. Do you have any additional comments or questions for our research team?

Lastly, we will send you your choice for a 20 dollar gift card from Amazon.com, Domino’s Pizza, Wendy's or Quiznos for participating in the phone interview. Which would you like? What address would you like us to mail the gift card to?

THANK YOU.
