# Supplementary material for: Impact of an Educational Comic to Enhance Patient-Physician–Electronic Health Record Engagement: Prospective Observational Study
Source: JMIR Hum Factors. 2021 Apr 28;8(2):e25054. doi: 10.2196/25054 (PMC8116991; doi:10.2196/25054)
Supplement: Multimedia Appendix 3 [file humanfactors_v8i2e25054_app3.docx]

**Multimedia Appendix 3: Patient perceptions of comic intervention: Post-visit survey results.**

|  | **Adult Sample**  **(n = 197)** | | | **Pediatric Sample**  **(n = 325)** | | |
| --- | --- | --- | --- | --- | --- | --- |
| ***TODAY, my physician...*** | **Disagree** | **Neutral** | **Agree** | **Disagree** | **Neutral** | **Agree** |
| 1) Made sure I could see the screen | 10.5% | 11.0% | **78.5%** | 5.0% | 4.0% | **91.0%** |
| 2) Made sure we could talk face to face | 3.1% | 4.1% | **92.8%** | 4.0% | 3.4% | **92.6%** |
| 3) Encouraged interaction with the computer | 14.9% | 17.0% | **68.1%** | 9.2% | 10.8% | **80.0%** |
| 4) Knew when to give full attention to me | 7.7% | 8.8% | **83.5%** | 3.9% | 10.6% | **85.5%** |
| 5) Used to computer to educate me | 13.2% | 25.2% | **61.6%** | 9.3% | 12.5% | **78.2%** |
| 6) Valued the computer | 7.7% | 20.0% | **72.3%** | 7.8% | 16.7% | **75.5%** |
|  | | | | | | |
| ***Compared to my last visit with this physician, TODAY...*** | **Disagree** | **Neutral** | **Agree** | **Disagree** | **Neutral** | **Agree** |
| 7) The physician used the computer to communicate more effectively | 7.3% | 25.8% | **66.9%** | 8.8% | 34.5% | **56.7%** |
| 8) The physician was less distracted by the computer | 12.0% | 26.1% | **61.9%** | 8.0% | 34.6% | **57.4%** |
| 9) I understood more because of education provided with the computer | 12.5% | 25.6% | **61.9%** | 10.5% | 34.3% | **55.2%** |
| 10) The physician made a better effort to involve me with the computer | 15.9% | 25.8% | **58.3%** | 10.6% | 34.3% | **55.1%** |
| 11) The physician was better about sharing the screen with me | 15.5% | 21.9% | **62.6%** | 10.2% | 31.2% | **58.6%** |
| 12) I was more satisfied with our relationship because of how they used the computer | 13.9% | 30.5% | **55.6%** | 12.1% | 41.3% | **46.6%** |
|  | | | | | | |
| ***Because of the comic...*** | **Disagree** | **Neutral** | **Agree** | **Disagree** | **Neutral** | **Agree** |
| 13) I asked to see the screen | 29.8% | 32.9% | **37.3%** | 41.0% | 32.9% | **26.1%** |
| 14) I asked to be more involved with the computer | 32.9% | 29.2% | **37.9%** | 38.6% | 31.5% | **29.9%** |
| 15) I felt more empowered about getting involved with the computer | 25.0% | 28.1% | **46.9%** | 24.8% | 31.2% | **44.0%** |
| 16) I felt more comfortable asking for the physician's full attention | 22.5% | 31.2% | **46.3%** | 25.6% | 36.2% | **38.2%** |
| 17) I am more likely to get involved with the computer in the future | 18.1% | 24.4% | **57.5%** | 17.4% | 28.1% | **54.5%** |
| 18) I think it's a good way to encourage involvement with the computer | 11.8% | 17.4% | **70.8%** | 7.4% | 21.0% | **71.6%** |
